# Wilkie's Syndrome following Chemotherapy: A Case Report and a Review of Literature

**DOI:** 10.1155/2022/7783074

**Published:** 2022-07-22

**Authors:** Nicholas J. Corsi, Ahmad A. Abu-Heija, Anand Kumar Ravi, Matthew P. Corsi, Murray N. Ehrinpreis

**Affiliations:** ^1^Wayne State University School of Medicine, Detroit, MI, USA; ^2^John D. Dingell V.A. Medical Center, Detroit Medical Center, Wayne State University, Detroit, MI, USA

## Abstract

Superior mesenteric artery (SMA) syndrome is a rare etiology of upper gastrointestinal obstruction. The measured angle between the SMA and the aorta is typically between 38 and 65° and maintained by mesenteric fat. Excessive fat loss can lead to intestinal obstruction due to an exaggerated acute angularity of the SMA, compressing the third part of the duodenum. We present a 22-year-old female with a history of aplastic anemia, status post bone-marrow transplant, who presented with intractable nausea and had confirmed SMA syndrome on CT angiography. Subsequently, the patient underwent nasogastric decompression and successful laparoscopic duodenojejunostomy.

## 1. Introduction

Superior mesenteric artery (SMA) syndrome, or Wilkie's syndrome, is a rare etiology of upper gastrointestinal obstruction, with potentially nonspecific symptoms on initial presentation. The prevalence of SMA syndrome in the general population is estimated at 0.013%–0.3% [1]. Normally, the measured angle between the SMA and the aorta is 38–65° maintained by mesenteric fat padding. The pathological mechanism of intestinal obstruction is due to an exaggerated acute angularity of the SMA, leading to compression of the third part of the duodenum between the SMA and aorta, caused by excessive fat loss. There are multiple predisposing factors that can increase the angularity, leading to duodenal compression, of which the most common is significant weight loss [1]. However, substantial weight loss and low BMI are not required for development of SMA syndrome. Gastroenterologists should be cognizant of other underlying medical disorders, previous surgeries, and psychological disorders that can heighten a patient's risk. Specifically, unexpected cases have been reported due to anorexia nervosa [2], spinal cord injury and kyphoscoliosis, and bariatric surgery [1]. However, there are limited cases reported of SMA syndrome subsequent to chemotherapy.

## 2. Case Presentation

**Figure 1:**
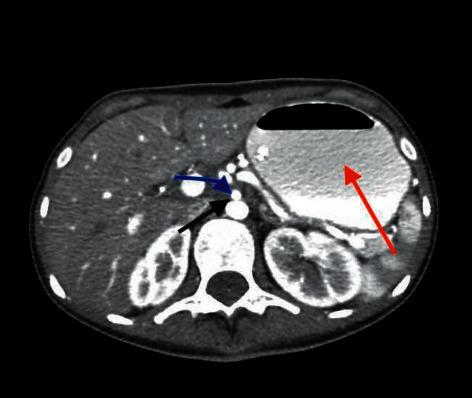
Axial CT abdomen showing compression of the third portion of the duodenum (black arrow) from the superior mesenteric artery (blue), with distention of the stomach (red arrow).

**Figure 2:**
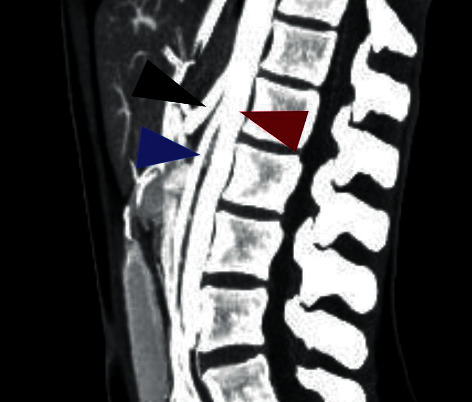
CT angiography revealing severe acute angularity of the SMA-aortic take off. Black arrow: celiac artery; maroon arrow: aorta; blue arrow: superior mesenteric artery.

**Figure 3:**
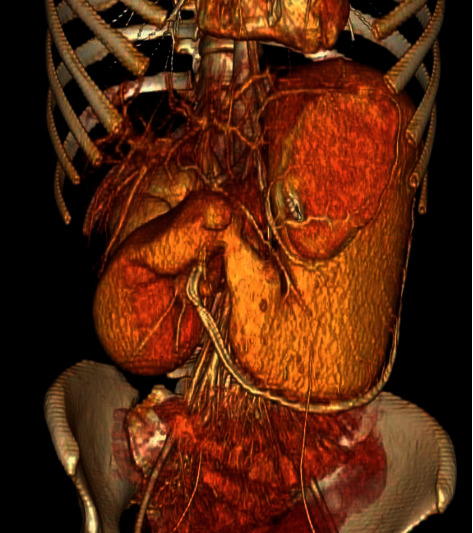
3D reconstructed CT angiography revealing severely distended stomach and first and second portion of the duodenum.

We present a 22-year-old female with no prior comorbidities who clinically manifested with lethargy, menometrorrhagia, and pancytopenia, and a subsequent diagnosis of aplastic anemia.She was scheduled for a stem-cell transplant, from a fully matched, unrelated donor. At time of initial medical oncology evaluation intake, she weighed 49.5 kg and was 160.7 cm tall (BMI = 19.2). Subsequently, she underwent a conditioning regimen of cyclophosphamide-fludarabine-antithymocyte globulin (Cy/Flu/ATG) prior to her allogenic bone-marrow transplant. She received all 5 days of scheduled fludarabine as well as 2 days of scheduled cyclophosphamide. On day 9 posttransplant, her weight continued to precipitously drop to 48.2 kg, to 47.9 kg, 46.6 kg, and 43.2 kg (on days 12, 14, and 50, respectively). She was drinking suboptimal amount of fluid (30–40 oz per day), and she reported no appetite and had minimal desire for food items she once enjoyed. She was refractory to antiemetics, as well as dronabinol for appetite stimulation. On consultation to gastroenterology, she complained of persistent nausea and vomiting episodes with any oral intake. Additionally, the only nutrition she was taking in regularly was 2 protein shakes with a total of 550 calories per shake. Finally, due to the intractable and intolerable nausea and vomiting, she presented to the emergency department (at a weight of 42.1 kg). Physical examination revealed peri-umbilical tenderness but was otherwise normal. Further history revealed a similar constellation of symptoms approximately 1 month prior, with a normal esophagogastroduodenoscopy, and resolution of symptoms with supportive care. During this hospitalization, however, she was initiated on total parenteral nutrition, due to malnutrition and inability to maintain >50% of caloric requirements by mouth. She was extremely fatigued, requiring even assistance to use the commode. During hospitalization, she continued to have asymptomatic sinus tachycardia, of which cardiology believed to be a physiologic response due to her volume-depleted state and acute dehydration. Her tachycardia improved with resolution of pain with vomiting and increasing physical strength. On readmission imaging, CT scan showed a massively distended stomach, first, and second portions of the duodenum, after collapse of the third portion of the duodenum as it traverses the SMA (Figure 1). CT-angiography was obtained revealing acute angulation of the SMA-aortic takeoff at approximately 20 degrees (Figures 2 and 3). A diagnosis of SMA syndrome was made. It was deduced that the chemotherapy-induced nausea, vomiting, and mucositis had led to appreciable weight loss and absence of the mesenteric fat pad, resulting with SMA syndrome. The patient underwent nasogastric decompression. By the day of surgical treatment, she dropped to 39.5 kg. Her nadir was 38.8 kg (BMI = 15) 4 days post-op. She underwent a laparoscopic duodenojejunostomy resulting in a successful bypass. Additionally, with surgical treatment and discontinuation of the chemotherapy, her weight gradually progressed to 47 kg. The patient was then followed for resolution of the preoperative symptoms. She demonstrated appropriate emptying of the duodenum and her subsequent follow up was uneventful.

## 3. Discussion

Very few cases of SMA syndrome postchemotherapy have been reported in the literature.  Table 1 offers a review. Each highlights the seemingly noteworthy complications in patients receiving treatment for malignancy, who may experience weight loss and severe emesis, which likely confound the presentation of the disease. Physicians should be wary of this rare etiology of duodenal obstruction and its clinical manifestations. Usually, the diagnosis can be confused with various motility-related or anatomic causes of duodenal obstruction. Albeit rare, a high index of suspicion should be employed for young patients presenting with signs and symptoms of intestinal obstruction with a history of weight loss, to allow inclusion of SMA (Wilkie's) syndrome in the differential diagnosis. Delayed diagnosis can lead to devastating outcomes, including death from complications related to electrolyte abnormalities or perforation. Furthermore, in patients who present with persistent emesis, SMA syndrome can precipitate hypovolemia, hypokalemia, and metabolic alkalosis. In order to confirm the diagnosis in a patient with symptoms suggestive of SMA symptoms, radiological studies are required, which may include an upper gastrointestinal (GI) barium studies alongside simultaneous angiography [3] or CT abdomen for noninvasive, enhanced anatomical detail. However, even with imaging, the diagnosis may be easily overlooked [4], as radiological findings do not always correlate with clinical findings [5]. Additionally, some patients may experience intermittent symptomatic compression of the duodenum, leading to a delay in diagnosis [1]. This was illustrated with our patient, who presented with similar symptoms 1 month prior to diagnosis, with no anatomic abnormalities on esophagogastroduodenoscopy. Depending on initial management, it is recommended that once symptom free and patency of the duodenum is achieved, patients begin a dietary regimen while cautiously monitoring clinically and biochemically for refeeding syndrome. Some suggest reversal of weight as initial therapy; however, this is based off of limited evidence. In one of the largest case series of SMA syndrome, the authors concluded that surgical management is superior to medical therapy with respect to success and recurrence, and surgery should be employed when conservative management fails, with lack of consensus on timing for surgical management [3]. Medical therapy consists of supportive care such as total parenteral nutrition or small-volume meals and postural changes [6]. Ultimately, in those who do not improve with conservative management, the surgical options include mobilization of ligament of Treitz (Strong's procedure) [7], gastrojejunostomy, and laparoscopic duodenojejunostomy. Of those options, duodenojejunostomy may provide the best results [8], which is reflected in the successful outcome in our patient.

In summary, physicians should be wary of this rare etiology of duodenal obstruction and its clinical manifestations. Albeit rare, a high index of suspicion should be employed for patients presenting with signs and symptoms of intestinal obstruction in the setting of chemotherapy, to allow inclusion of SMA syndrome in the differential.

## Figures and Tables

**Table 1:** Review of reported cases of SMA syndrome following chemotherapy.

First author, year	Age (sex)	Chemotherapeutic agent	Cancer	Associated symptoms	Weight loss (kg)	Imaging	Treatment
Ushiki, 2012 [9]	61 (M)	Cisplatin	Squamous cell carcinoma, lung	Anorexia, nausea, vomiting	7	CT abdomen, upper GI series, abdominal X-ray	Tubal feeding; weight gain
Chowdhary, 2015 [10]	81 (M)	Carboplatin	Mesothelioma	Epigastric pain, nausea, vomiting	18	CT abdomen, upper GI series, esophagogram	Conservative; weight gain
Irei, 2016 [11]	∼60 s (M)	Neo-adjuvant chemotherapy	Esophageal	Postprandial abdominal pain	6	CT and upper GI series	Roux-en-Y bypass of gastrostomy and jejunostomy
Lippl, 2002 [12]	31 (M)	Palliative chemotherapy	Colorectal cancer	Recurrent vomiting	7	CT abdomen, digital fluoroscopy, esophagogastroduodenoscopy	Parenteral nutrition; weight gain
Bang, 2009 [13]	57 (F)	Palliative chemotherapy	Colon cancer with peritoneal seeding	Post-prandial abdominal pain, nausea	6	CT abdomen and small bowel series	Adhesiolysis and duodenojejunostomy
Girotra, 2013 [14]	60 (F)	Chemotherapy	Breast cancer	Post-prandial pain, vomiting, nausea	36	CT abdomen, barium swallow, esophagogastroduodenoscopy	Conservative; weight gain

## Data Availability

Data are readily available and readers may access the data supporting the conclusions of the study by directly emailing the corresponding author.
